# SKA2-mediated transcriptional downregulation of the key enzyme of CoQ_10_ biosynthesis PDSS2 in lung cancer cells

**DOI:** 10.7150/jca.79058

**Published:** 2023-01-16

**Authors:** Xing Xia, Chuntao Tao, Kailong Du, Peixin Meng, Lanyue Hu, Dong Cheng, Xianjun Liu, Youquan Bu, Xiaoyan Fan, Quanmei Chen

**Affiliations:** 1 Department of Biochemistry and Molecular Biology, College of Basic Medical Sciences, Chongqing Medical University, Chongqing 400016, China.; 2 Molecular Medicine and Cancer Research Center, College of Basic Medical Sciences, Chongqing Medical University, Chongqing 400016, China.; 3 Department of Basic Medical Sciences, Taizhou University, Taizhou, Zhejiang 318000, China.

**Keywords:** SKA2, PDSS2, transcription regulation, lung cancer

## Abstract

Lung cancer is the leading cause of cancer-associated mortality worldwide. SKA2 is a novel cancer-associated gene that plays critical roles in both cell cycle and tumorigenesis including lung cancer. However, the molecular mechanisms underlying its implication in lung cancer remains elusive. In this study, we first analyzed the gene expression profiling after SKA2 knockdown, and identified several candidate downstream target genes of SKA2, including PDSS2, the first key enzyme in CoQ_10_ biosynthesis pathway. Further experiments verified that SKA2 remarkably repressed PDSS2 gene expression at both mRNA and protein levels. Luciferase reporter assay showed that SKA2 repressed PDSS2 promoter activity through its Sp1-binding sites. Co-immunoprecipitation assay demonstrated that SKA2 associated with Sp1. Functional analysis revealed that PDSS2 remarkably suppressed lung cancer cell growth and motility. Furthermore, SKA2-induced malignant features can be also significantly attenuated by PDSS2 overexpression. However, CoQ10 treatment showed no obvious effects on lung cancer cell growth and motility. Of note, PDSS2 mutants with no catalytic activity exhibited comparable inhibitory effects on the malignant features of lung cancer cells and could also abrogate SKA2-promoted malignant phenotypes in lung cancer cells, highly suggesting a non-enzymatic tumor-suppressing activity of PDSS2 in lung cancer cells. The levels of PDSS2 expression were significantly decreased in lung cancer samples, and lung cancer patients with high expression of SKA2 and low expression of PDSS2 displayed remarkable poor prognosis. Collectively, our results demonstrated that PDSS2 is a novel downstream target gene of SKA2 in lung cancer cells, and the SKA2-PDSS2 transcriptional regulatory axis functionally contributes to human lung cancer cell malignant phenotypes and prognosis.

## Introduction

Lung cancer is the leading cause of cancer-related deaths worldwide, accounting for 2 million new cases and 1.76 million deaths per year 1. Over the past several years, surgical resection has remained the main approach of treatment for patients with lung cancer and its 5-year survival rate was found to be about 20% 2, 3. Therefore, searching for diagnostic biomarkers and effective therapeutic strategies is a pressing need in lung cancer.

SKA2 (spindle and kinetochore associated complex subunit 2) is a novel identifed gene implicated in cell cycle regulation and tumorigenesis 4. SKA2 protein is a crucial component of SKA complex composed of SKA1, SKA2 and SKA3, required to stabilize kinetochore-microtubule interactions 5. Furthermore, the study of the crystal structure of the SKA complex suggests that SKA2 function as a scaffold to stabilize the complex 6. RNAi-mediated knockdown of SKA2 retarded the cell cycle progress during metaphase 7. Significant upregulation of SKA2 is found in multiple cancer cells, and it accelerates carcinoma cell proliferation, migration and invasion 8-11. Whereas downregulation of SKA2 is associated with depression and suicidal ideation 4, the expression of SKA2 is important for human health to maintain its normal constitutive levels. And SKA2 expression levels in lung cancer differ amongst histological types and differentiation stages 12. Moreover, strong expression of SKA2 is correlated with poor prognosis both in lung cancer and breast cancer 12, 13. Taken together, aberrant expression of SKA2 is associated with tumor progression. Additionally, the transcriptional regulatory mechanisms of SKA2 gene were investigated, previous studies revealed that some transcription factors such as NF-Y (nuclear transcription factor Y), CREB (cyclic AMP responsive element-binding protein), NF-κΒ, and p53, non-coding RNAs including miR-301, miR-141 and Circ_0008039, and Hedgehog pathway member GLI1/2, are implicated in dysregulation of SKA2 expression and contributed to tumor development 11, 14-18. Nevertheless, downstream genes regulated by SKA2 in cancer cells are still vacant. Previous study has shown that SKA2 co-localizes and interacts with glucocorticoid receptor (GR), and enhancing the cancer cell proliferative response to IGF-1 exposure 8, suggesting that SKA2 should be capable of regulating its target genes to augment cancer cell progression. Therefore, target genes regulated by SKA2 are needed to be explored for understanding the underlying mechanism of SKA2 promoting tumor progression.

In the present study, we for the first time found that PDSS2 (prenyl diphosphate synthase subunit 2), the first key enzyme in CoQ_10_ biosynthesis pathway, is a novel downstream transcriptional target gene of SKA2. PDSS2 displayed tumor-suppressing activity and could abrogate the SKA2-induced cell proliferation and motility in lung cancer cells. The expression of this novel transcriptional regulatory axis of SKA2 and PDSS2 also has prognostic value for lung cancer patients, highly suggesting its substantial contribution to human lung cancer progression.

## Materials and Methods

### Cell culture and CoQ_10_ treatment

Human lung cancer cell lines, A549 and H1299, were purchased from Chinese Academy of Sci-ences Shanghai cell bank (Shanghai, China). Cells were cultured in DMEM F-12 (Hyclone, Utah, USA) for A549 or RPMI 1640 for H1299 medium supplemented with 10% fetal bovine serum (Hyclone, Utah, USA), 1% penicillin and streptomycin. Cells were incubated at 37°C in a humidified atmosphere with 5% CO2, and then cells in exponential growth phase (approximately 70-80% con-fluence) were used in experiments. CoQ_10_ powder was purchased from MCE Company (No. HY-N0111). 1mg of CoQ_10_ powder was weighed and placed in 1.5mL centrifuge tube, and then adding 115.8 μL Dimethylformamide (N' n-dimethylformamide N', N-, DMF) to dissolve at 37℃ water bath and ultrasound (frequency: 40KHZ) for 10min. CoQ_10_ (50 μM, 100 μM) were added, respectively, indicated time after CoQ_10_ treatment, cells were subjected to further analysis.

### RNAi and overexpression

Overexpression plasmids, pcDNA3.0-SKA2 and pcDNA3.0-Flag-PDSS2, were constructed using the seamless cloning kit (Novorec®PCR NR001, Novoprotein, Shanghai, China) and maintained in our laboratory. Three overexpression constructs of PDSS2 mutants including pcDNA3.0-PDSS2Q322X, pcDNA3.0-PDSS2S382L, pcDNA3.0-PDSS2-Del2 were generated using a site-directed mutagenesis kit (Toyobo, Japan) on the basis of the wild type PDSS2 construct according to the manufacturer's instruction. The primer sequences were listed in the Table 1, the sequences of the cloned DNA fragments were confirmed by DNA sequencing. RNAi-mediated knockdown was performed according to our previous method 12, 19. Briefly, the nucleotide sequences of siRNAs against SKA2 and shRNA against PDSS2 were shown in Table 1, and were chemically synthesized by Shanghai GenePharma (Shanghai, China). The oligonucleotides of shRNA were annealed and then cloned into the vector LVRH1GP to generate LVRH1GP-shPDSS2 recombinant plasmids. Prior to transfection, cells were seeded at a density 2×10^5^ cells/6-well cell culture plate and then incubated overnight. Cells were applied for transient transfection with indicated plasmids using Neofect DNA® transfection reagent (Neofect biotech, Beijing, China) according to the manufacture protocol. The siRNAs were transfected into cells using Lipofectamine RNAiMAX reagent (Invitrogen, Carlsbad, CA, USA) according to the manufacturer's instructions. Cells were then collected and subjected to further analysis. The experiments were repeated at least three times.

### Identification of differentially expressed genes and enrichment analysis

The gene expression dataset (E-MEXP-875) was downloaded from the European Bioinformatics Institute (EMBL-EBI) (https://www.ebi.ac.uk/). Four SKA2 knockdown samples and two corresponding control samples were used for differential gene analysis by R, FoldChange, which was used for the screening of differentially expressed genes, the threshold: |log2FC| ≥ 1. To better explore the biological significance of differentially expressed genes, the DAVID v6.8 19 was utilized to conduct gene ontology analysis and pathway enrichment analysis. Over-presented transcriptional factors binding site motifs of differential expressed genes were scaned by Pscan 20.

### Quantitative RT-PCR

Total RNA was isolated from cells using the Total RNA Kit I (Omega Bio-Tek). Total RNA was quantified using the Nanodrop 2000 spectrophotometer (Thermo Scientific). A total of 500 ng of RNA was reverse-transcribed using the Total RNA Kit I (Omega Bio-Tek) according to the manufacturer's instructions and diluted in nuclease-free water. Quantitative real-time PCR (qRT-PCR) was conducted using SYBR® Premix Ex Taq (Perfect Real Time, Takara Tokyo, Japan) according to the manufacture protocol 21. TissueScan lung cancer panels (HLRT103) containing prepared cDNAs were purchased from OriGene (Rockville, MD). The sequences of the primers were displayed in Table 2. The relative quantification was calculated using the 2^-ΔΔCT^ relative quantification method.

### Co-immunoprecipitation (CoIP) and Western blotting

Co-immunoprecipitation (CoIP) assays were carried out as described previously 22. Briefly, H1299 cells were transiently co-transfected with the pcDNA3.0-Flag-SKA2, pcDNA3.0-HA-Sp1 expression constructs. Whole cell extracts were prepared and precleared with Protein A/G Magnetic Beads for 1 h at 4 °C, and then incubated with primay anti-HA antibody (Bimake) and normal rabbit IgG (Cell Signaling Technology) overnight at 4 °C. The antigen-antibody complexes were then captured by Protein A/G Magnetic Beads. The immunoprecipitates were separated by a magnetic separator, and finally subjected to immunoblotting analysis with the indicated antibodies.

Western blotting was conducted as described previously 23. Briefly, Cell monolayers were washed twice with PBS, and then harvested and lysed with ice-cold RIPA buffer supplemented with protease inhibitor Cocktail (Biotool, Houston, TX, USA). The total proteins were measured using the bicinchoninic acid (BCA) protein assay kit (Thermo Scientific, Beijing, China) and then subjected to SDS-PAGE and immunoblotting. The antibodies used for immunoblotting included those raised against target protein SKA2, PDSS2 and CoQ10B. The primary antibodies were anti-SKA2 (Invitrogen, Carlsbad, CA, USA, No.PA5-20818, dilution 1:500, with secondary anti-rabbit antibodies), anti-PDSS2 (Santa Cruz Biotechnology, Santa Cruz, CA, USA, No. sc-515137, dilution 1:200, with secondary anti-mouse antibodies) and anti-CoQ10B (Abcam, Cambridge, MA, USA, No. ab41997, dilution 1:1500, with secondary anti-rabbit antibodies), Antibody binding was detected by enhanced chemiluminescence (ECL, Bio-Rad Laboratories, Hercules, CA, USA). GAPDH was used as an internal control.

### Luciferase reporter assay

The promoter reporter vectors PDSS2-P2031 (-1768/+263), PDSS2-P764 (-501/+263), PDSS2-P464 (-201/+263), PDSS2-P202(-201/+1), and a variety of Sp1 binding sites mutant vectors of PDSS2-P202 were constructed previously and maintained in our laboratory 24. The luciferase reporter assay was conducted as described previously in detail 12. Briefly, equal number of cells was seeded into 12-well culture plates in triplicate, and then transfected with the corresponding plasmids when the confluence of cells reaches 50-60%. Forty-eight hours after transfection, the luciferase activities were determined using Dual-Luciferase assay system (E1960, Promega, USA) and fold activity were utilized to indicate the promoter activities.

### Cell proliferation and migration assays

Cell proliferation was measured using CCK8 (Dojindo, Tokyo, Japan), and JuLI™ Stage Real-Time Cell History Recorder (NanoEntek, Seoul, South Korea), the details were described previously 23, 25, 26. The live cell analyzer monitored cell confluence by recording cell images at 2-hour intervals. On the first day, 2 × 10^5^ cells were seeded in each well. During log growth phase, monitored continuously for 54 h. Cell numbers were counted by CellDrop FL Fluorescence Cell Counter (Devovix, USA).Would healing, and transwell migration analysis were performed as described previously 23, 25. Motility experiments were done according to our previous research 26. Briefly, cells were harvested and resuspended at a density of 1 × 10^4^ cells/mL in 6-well culture plates, and then images were taken continuously using JuLI™ Stage Real-Time Cell History Recorder (NanoEntek, Seoul, South Korea) for 12 hours with an interval of 15 min. The cell motility ability was quantified via Image-pro.

### Survival analysis in lung cancer by Kaplan-Meier plot

The potential impact of related gene expression on overall survival (OS) in lung adenocarcinoma patients was analyzed. Graphpad software was used to generate Kaplan-Meier plots for 130 lung cancer patients in the GSE13213 dataset. Based on the cut-off value of the median survival time, lung cancer patients were divided into PDSS2-high/SKA2-low (n=29), PDSS2-low/SKA2-low (n=44), PDSS2-high/SKA2-high (n=26) and PDSS2-low/SKA2-high (n=18) groups, the Kaplan-Meier survival curve was generated that predicts cases with low or high risk.

### Statistical analysis

Statistical analyses method was described previously in detail 22. In brief, the SPSS 16.0 statistical software package (SPSS Inc, Chicago, USA) was applied for statistical analyses, and results were shown as mean ± SD. *P* values less than 0.05 were considered statistically significant, *P < 0.05, **P < 0.01, ***P < 0.001.

## Results

### Identification of differentially expressed genes after SKA2 knockdown

**Figure 1:**
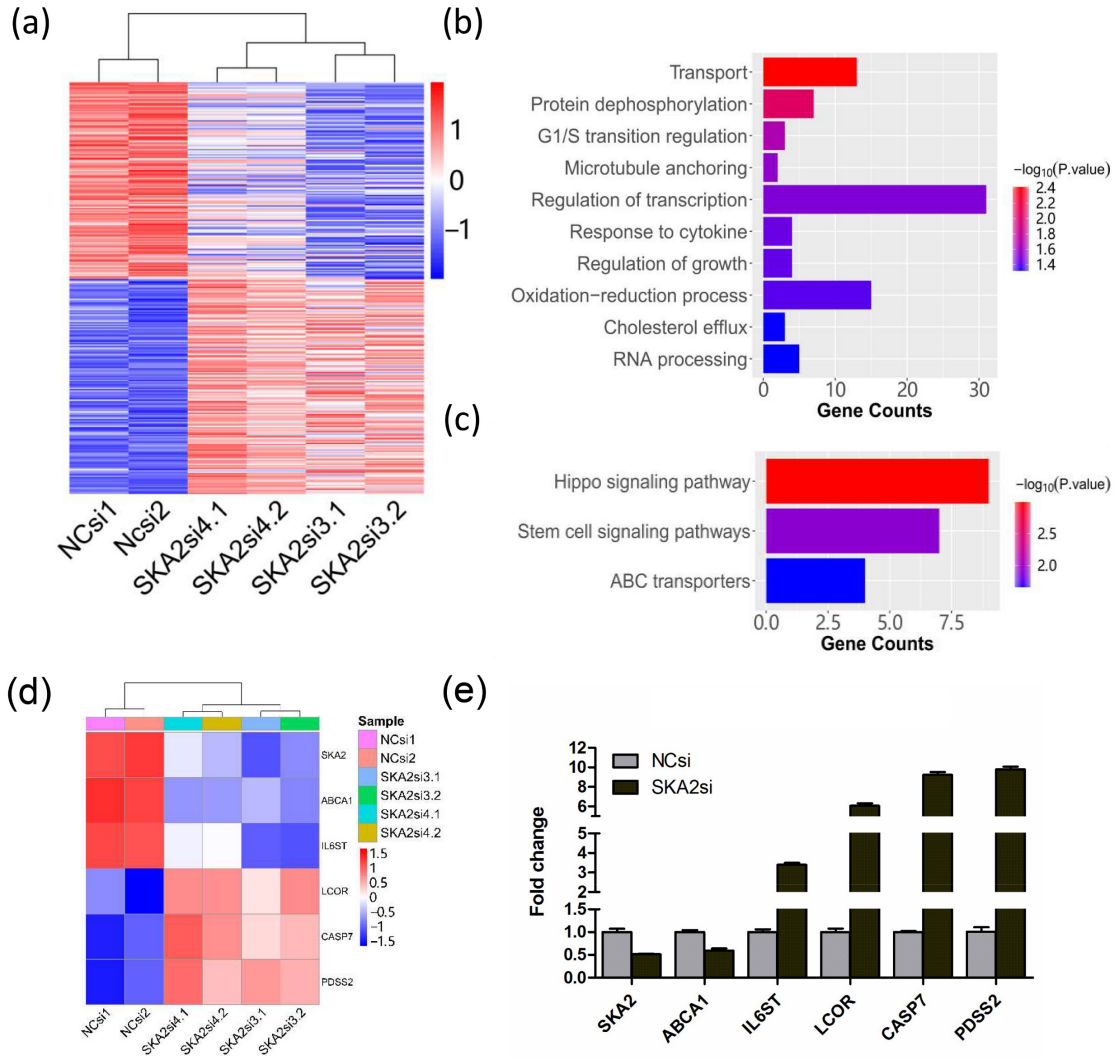
Identification of differentially expressed genes after RNAi-mediated SKA2 knockdown. (**a**) Heatmap of differentially expressed genes after SKA2 knockdown with two independent siRNAs against SKA2 (SKA2si3 and SKA2si4). NCsi denotes negative control siRNA. (**b**) GO enrichment and (**c**) KEGG pathway analysis of the differentially expressed genes after SKA2 knockdown compared with negative control siRNA group. (**d**) Heatmap of six differentially expressed genes after SKA2 knockdown. (**e**) Expression verification of the six genes by qRT-PCR.

To identify the potential downstream target genes regulated by SKA2, we first analyzed a previously published gene expression profiling data of SKA2 knockdown in human lung cancer cell line A549 (E-MEXP-875). For this microarray dataset, two independent siRNAs against SKA2 were used to knockdown SKA2 and avoid off-target effects. We performed differentially expressed gene analysis between the control and SKA2 knockdown groups. The results showed that there were 175 up-regulated genes and 199 down-regulated genes (Figure 1a). The GO analysis revealed that the differentially expressed genes were mainly enriched in transport, protein dephosphorylation, G1/S transition regulation, microtubule anchoring, regulation of transcription, response of cytokine, regulation of growth, oxidation-reduction process, cholesterol efflux, and RNA processing (Figure 1b). The pathway enrichment analysis showed that the differentially expressed genes were mainly implicated in hippo signaling pathway, stem cell signaling pathway and ABC transporters as shown in Figure 1c.

Furthermore, five differentially expressed genes implicated in important signaling pathways and metabolism including ABCA1, IL6ST, LCOR, CASP7 and PDSS2 were selected (Figure 1d). We then performed RNAi knockdown experiment in A549 cells. Quantitative RT-PCR experiment analysis verified that the expression of SKA2 was successfully silenced, and the five selected genes were indeed remarkably altered after SKA2 knockdown (Figure 1e).

### SKA2 inhibits endogenous PDSS2 expression in lung cancer cells

**Figure 2:**
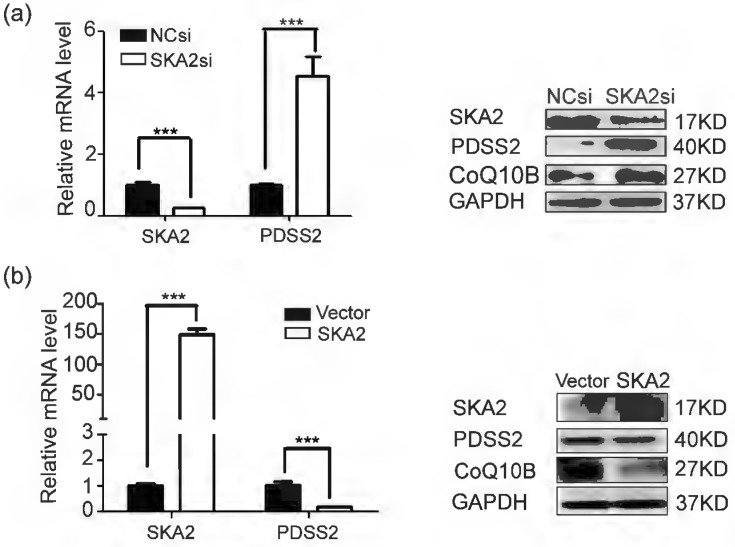
SKA2 inhibits PDSS2 expression in lung cancer cells. (**a**) Knockdown of SKA2 caused PDSS2 upregulation. A549 cells were transiently transfected with negative control siRNA, SKA2 siRNA. Quantitative RT-PCR and western blotting were then performed to detect the expression of indicated genes. (**b**) Overexpression of SKA2 caused PDSS2 downregulation. A549 cells were infected with the empty control and SKA2 overexpression vector. Quantitative RT-PCR and western blotting were then performed to detect the expression of indicated genes. GAPDH was used as an internal control.

To determine whether PDSS2 is bona fide downstream target of SKA2. RT-PCR and Western blotting results showed that the expression of PDSS2 was significantly increased at both mRNA and protein levels after SKA2 knockdown (Figure 2a). In contrary, exogenous overexpression of SKA2 in A549 cells remarkably restrained PDSS2 expression (Figure 2b). These findings verified that SKA2 suppress the expression levels of PDSS2. Considering that PDSS2 is one of the rate-limiting enzymes in CoQ_10_ biosynthesis 27-29, and CoQ10B proteins required for CoQ_10_ function in respiration 30, we thus detected the CoQ10B protein levels after modulating SKA2 in A549 cells. As shown in Figure 2b, CoQ10B protein levels were strongly upregulated by SKA2 knockdown, and significantly reduced by ectopic expression of SKA2. The results suggested that SKA2 might regulate the biosynthesis and function of CoQ_10_ in cells.

### SKA2 suppresses PDSS2 promoter activity

**Figure 3:**
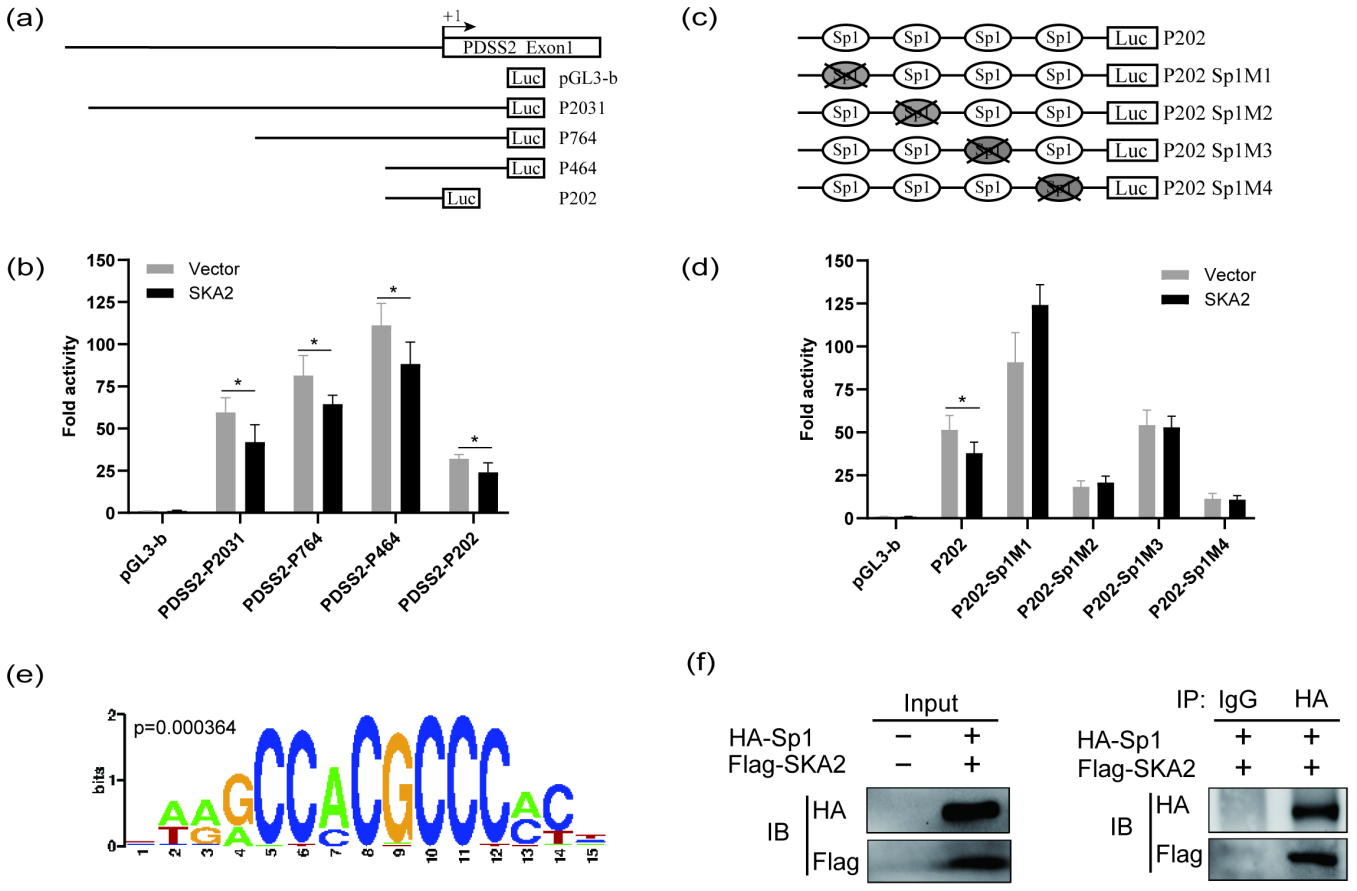
SKA2 inhibits the promotor activity of PDSS2. (**a**) Schematic diagram of the PDSS2 gene promoter reporter structure. +1 is indicated to the position relative to the major transcriptional initiation site of PDSS2. (**b**) Overexpression of SKA2 inhibits PDSS2 promoter activity in A549 cells. (**c**) Schematic diagram of site-directed mutagenesis of Sp1 binding sites in the PDSS2 core promoter region. The four potential Sp1 binding sites are marked as ovals. The indicated point mutation is denoted by a cross. (**d**) Overexpression of SKA2 is unable to reduce the luciferase activities of PDSS2 core promoter with Sp1 binding site mutation. The promoter activity was presented by Fold activity obtained by dividing the luciferase correction value of the experimental group by the empty vector pGL3-basic group. All experiments were performed in triplicates. *p<0.05. (**e**) The transcriptional factors binding sites of differentional genes are analyzed by Pscan, Sp1 bining site was enriched. (**f**) CoIP assays demonstrate the interacton of SKA2 and Sp1. Immunoprecipitation (IP) was executed with anti-HA antibody directed against HA-tagged Sp1and immunoprecipitation Blotted (IB) with anti-Flag antibody recognizing Flag-tagged SKA2.

To further confirm that SKA2 inhibits PDSS2 transactivation, the luciferase reporter assay was performed. We previously have established four luciferase reporter constructs with different PDSS2 gene promoter fragments, namely P2031 (-1768/+263), P764 (-501/+263), P464 (-201/+263), and P202 (-201/+1) 24, as schematically shown in Figure 3a. Hence, SKA2 overexpression plasmids were co-transfected with the indicated four PDSS2 promoter luciferase reporters in A549 cells, respectively. The luciferase reporter assay showed that exogenous overexpression of SKA2 dramatically reduced the luciferase activity of all of the four reporters with different extents in comparison with the empty vector transfected group (Figure 3b). The findings confirmed that SKA2 repressed PDSS2 transactivation. Our previous study has shown that Sp1 directly binds to the PDSS2 core promoter region (P202 (-201/+1)), and then downregulates the PDSS2 expression 24. Therefore, we determined whether SKA2-mediated PDSS2 downregulation depends on Sp1. Disruption each of the four Sp1 binding sites in PDSS2 core promoter region by point mutation generated four corresponding luciferase reporters, marked as P202M1, P202M2, P202M3, and P202M4, respectively (Figure 3c). As displayed in Figure 3d, disruption of each of the four Sp1 binding sites abrogated the inhibitory effect of SKA2 on PDSS2 promoter activity (Figure 3d). Our findings suggested that SKA2 repressed PDSS2 transcription through the Sp1-binding sites in PDSS2 core promoter. The transcriptional factors binding site motifs of the differential expressed genes after SKA2 knockdown were scaned using Pscan. The results revealed that Sp1 binding sites were significantly enriched in the promoter regions of the differentially expressed genes including PDSS2, IL6ST, CASP7 etc. (Figure 3e, Figure 1e). Indeed, Furthermore, CoIP assay indicated that SKA2 associated with Sp1 (Figure 3f). Nonetheless, more work is needed to reveal whether SKA2 directly interacts with Sp1.

### PDSS2 represses lung carcinoma cell proliferation and motility

**Figure 4:**
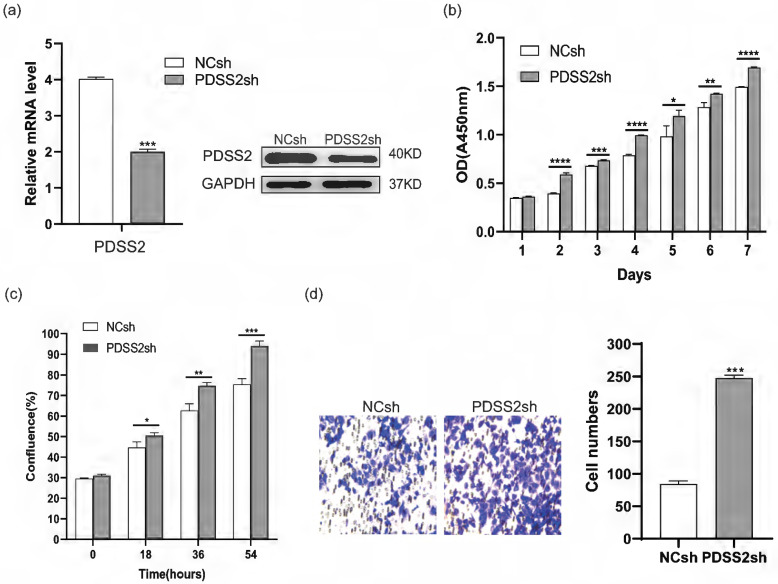
Depletion of PDSS2 represses lung cancer cell proliferation and migration. (**a**) PDSS2 knockdown. A549 cells were transfected with negative control shRNA (NCsh) and PDSS2 shRNA (PDSS2sh) vector. Two days after transfection, total RNA was isolated and cDNA was synthesized by reverse transcription. The PDSS2 gene expression was determined by qRT-PCR (left panel) and Western blotting (right panel). (**b**) PDSS2 decreases cell proliferation. Cell proliferation was detected in PDSS2-knockdowned cells at indicated times by CCK8 assay. (**c**) Continuous monitoring of cell growth by JULI Stage real-time cell history recorder. (**d**) Transwell migration assay.

**Figure 5:**
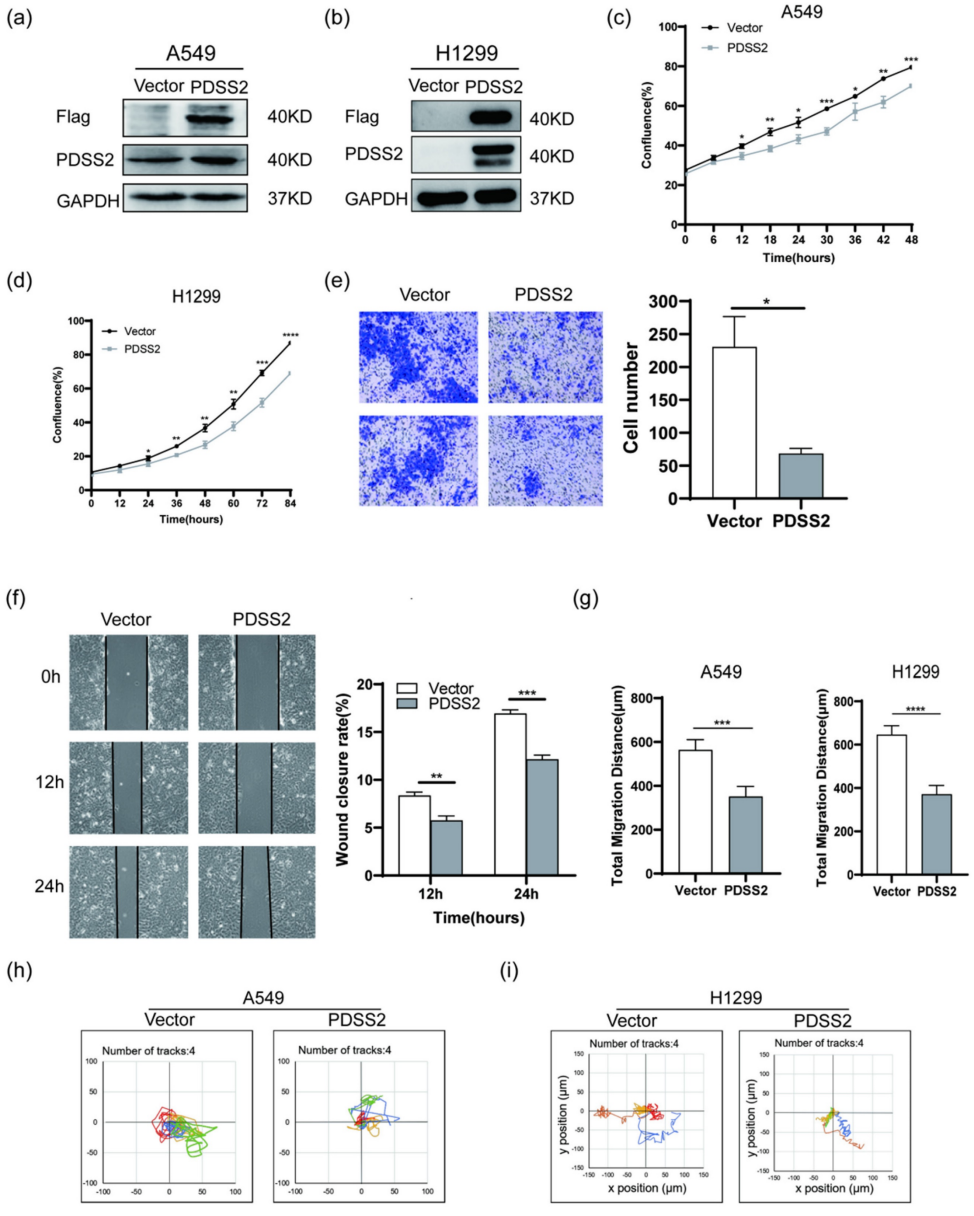
PDSS2 overexpression suppresses lung cancer cell proliferation and motility. (**a,b**) Exogenous overexpression of PDSS2. A549 and H1299 cells were transfected with PDSS2 overexpression vector or empty vector alone. After 48 hours, cell lysates were prepared, immunoblotting was conducted using antibodies against Flag, PDSS2 and GAPDH (loading control). (**c,d**) Cell growth monitoring. The growth of A549 and H1299 cells were continuously monitored by JuLI Stage Live Cell Imaging System. (**e**) Transwell migration assay in A549 cells. (**f**) Wound healing assay in A549 cells. PDSS2 overexpression plasmids and control vector were transiently transfected into A549 cells, respectively. Wound healing assay showed that overexpression PDSS2 repressed A549 cells motility. (**g, h,i**) Cell motility assay. Cell motility of A549 and H1299 was captured respectively every 15 minutes for 12 hours using JULI Stage real-time cell history recorder. Motile trajectory, mean total migration distances and mean migration speeds are presented.

We next investigated whether PDSS2 plays tumor-suppressing role in lung cancer cells. Endogenous PDSS2 expression was remarkably reduced by shRNA-mediated PDSS2 knockdown in A549 cells (Figure 4a). Both CCK-8 assay and live cell monitoring results showed that knockdown of PDSS2 promoted lung cancer cell growth (Figures 4b, 4c). Cell motility assay showed that knockdown of PDSS2 increased lung cancer cell migration capability (Figure 4d). Moreover, exogenous overexpression of PDSS2 was achieved by transiently transfecting PDSS2 overexpression plasmids into A549 and H1299 cells (Figures 5a, 5b). The results showed that forced overexpression PDSS2 resulted in significant decrease in lung cancer cell growth (Figures 5c,5d). In addition, Cell motility assay demonstrated that overexpression of PDSS2 also remarkably inhibited lung cancer cell motility (Figures 5e-5i). Previous study also found that PDSS2 significantly inhibits colony formation and causes cell death through apoptotic pathways in H1299 cells 31. Taken together, these results suggested that PDSS2 has significant tumor-suppressing activity in lung cancer cells.

### Functional implication of SKA2-PDSS2 regulatory axis in lung cancer

**Figure 6:**
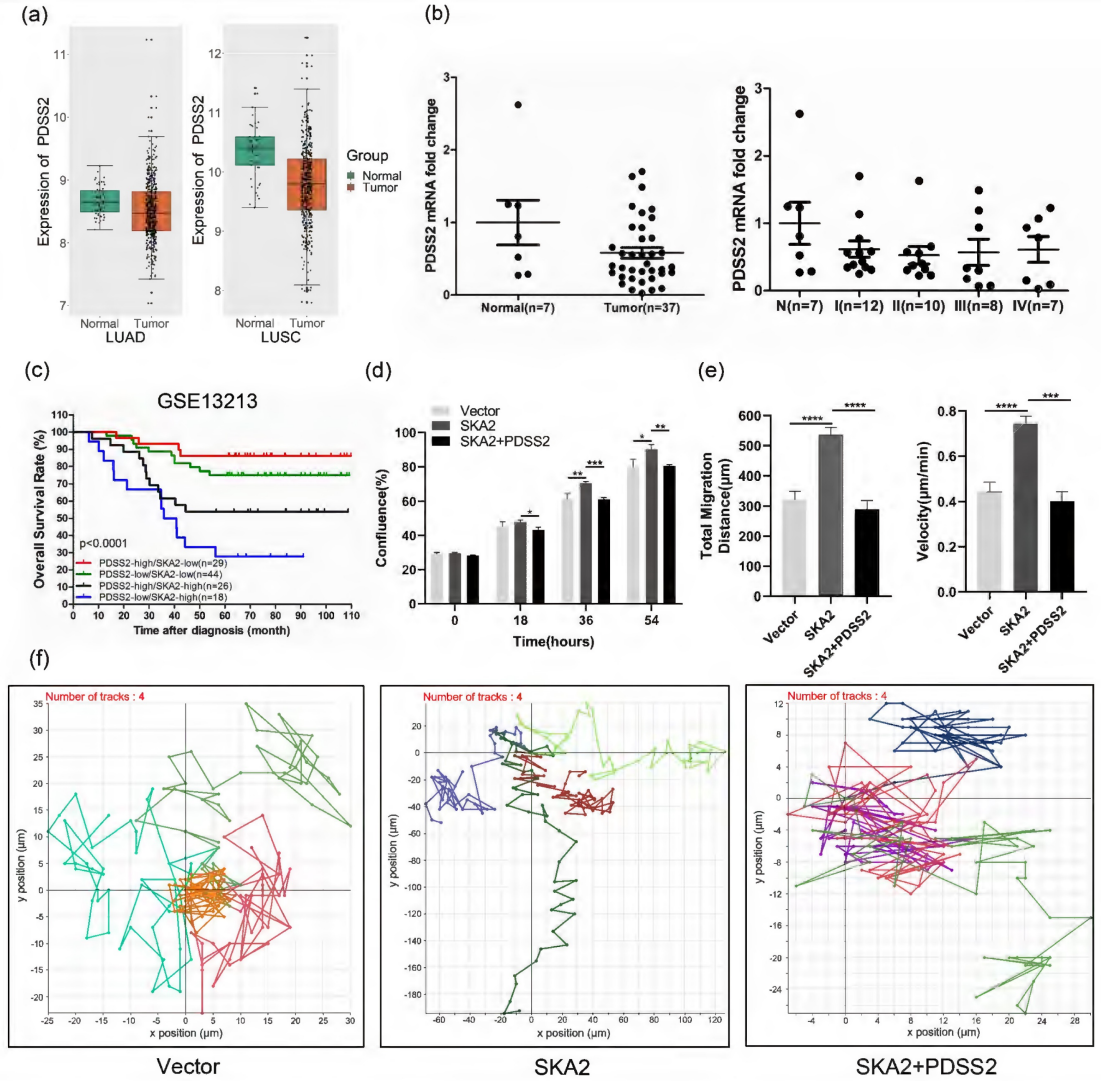
Functional implication of SKA2-PDSS2 axis in lung cancer. (**a**) Boxplots of PDSS2 expression in TCGA lung cancer dataset. (**b**) PDSS2 expression levels were analyzed with qRT-PCR using OriGene TissueScan lung cancer panels (HLRT103). The stages were divided into I, II, III, and IV. “N” means normal. The wide bars denote the mean levels of PDSS2, while the narrow ones represent standard deviation away from the mean. (**c**) Survival analysis in relation to SKA2 and PDSS2 expression in lung cancer. Kaplan-Meier plot of overall survival of lung cancer patients in the GSE13213 dataset, the p-values were calculated using Log-rank test. (**d**) PDSS2 abrogates SKA2-promoted lung cancer cells growth. Cells were transfected with indicated plasmids and cell growth was continuously monitored by JULI Stage Real-time Cell History Recorder. (**e, f**) Cell motility analysis. Cell motility was also captured every 15 minutes for 12 hours by the aforementioned instrument. Motile trajectory, mean total migration distances and mean migration speeds are presented.

Furthermore, we analyzed the expression of PDSS2 and its clinical significance in lung cancer. Bioinformatic analysis of the TCGA dataset revealed that PDSS2 expression was significantly downregulated in lung cancer tissues relative to normal lung tissues (Figure 6a). Quantitative RT-PCR analysis further confirmed the downregulation of PDSS2 and its association with the clinical stage in lung cancer tissues (Figure 6b). Of note, PDSS2 and SKA2 were associated with clinical outcome in that the worst overall survival was observed in lung cancer patients with high expression of SKA2 and low expression of PDSS2 while the best outcome shown in patients with overexpressed PDSS2 and low expression of SKA2 (Figure 6c). These findings highly suggested that the SKA2-PDSS2 axis might serve as a potential prognostic and diagnostic biomarker in lung cancer.

### SKA2-induced malignant features can be abrogated by PDSS2 overexpression

We determined whether SKA2-induced lung cancer cell proliferation and migration depends on its repression of PDSS2 expression. The results showed that overexpression of SKA2 alone increased lung cancer cell growth and migration, and these effects were completely abrogated by overexpression of PDSS2 (Figures 6d-6f). Taken together, these findings strongly suggested that SKA2 accelerates lung cancer cell development through repression of PDSS2.

### CoQ_10_ treatment shows no obvious effects on lung cancer cell growth and motility

**Figure 7:**
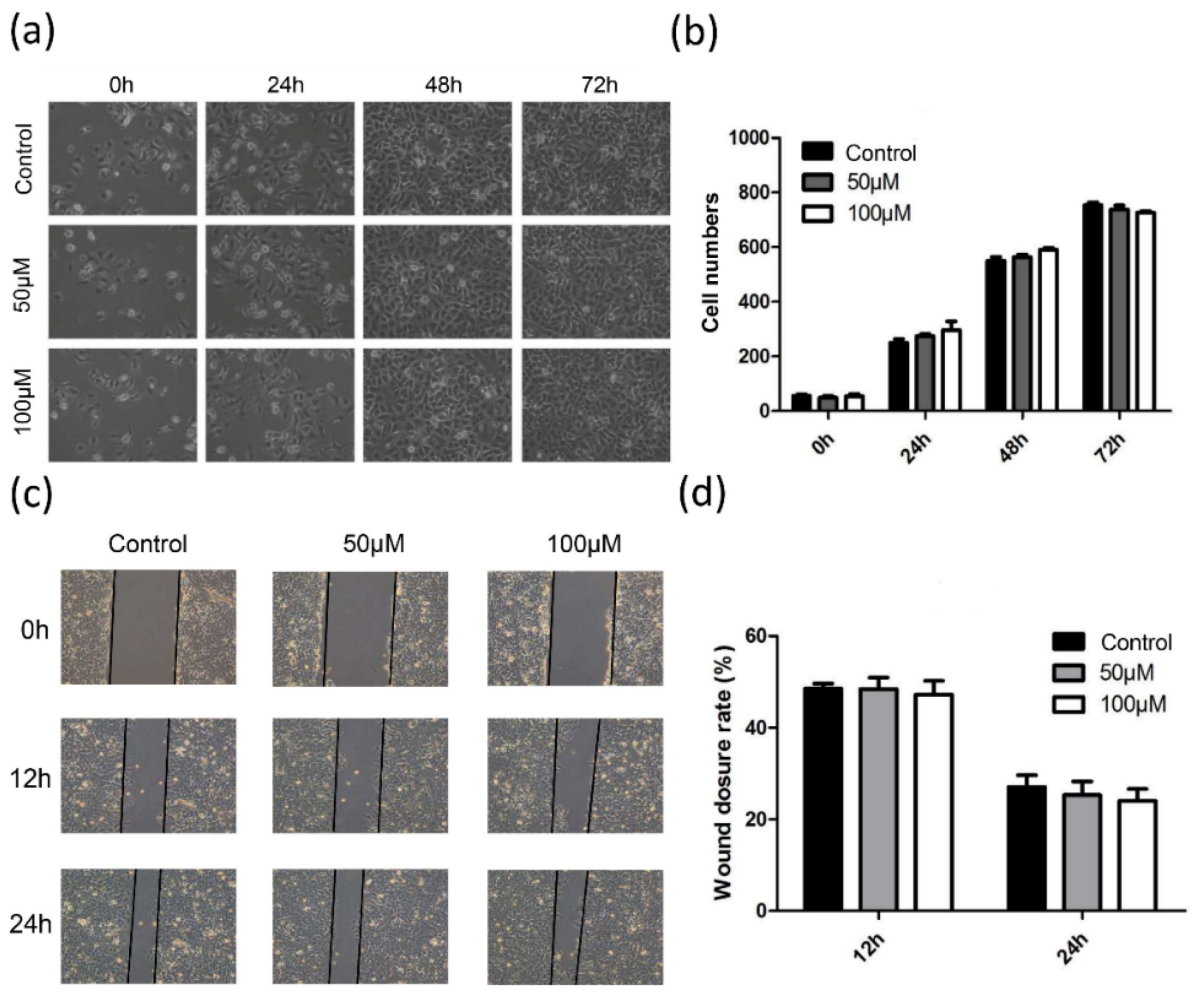
CoQ_10_ exerted no obvious effects on lung cancer cell growth and motility. (**a**) CoQ_10_ was added into A549 cells at the indicated concentrations, respectively, and then photographed under microscope at indicated time. (**b**) The number of cells were counted using ImageJ software. (**c**) Migration ability of A549 cells treated with CoQ_10_ was determined by wound healing assay (Original magnification: ×100). (**d**) The scratch width was measured using ImageJ software.

As PDSS2 is the rate-limiting enzyme for CoQ_10_ biosynthesis and the levels of CoQ10B was also suppressed by overexpression SKA2, we then decided to examine the effect of exogenous CoQ_10_ treatment on lung cancer cell phonotypes. To our surprise, the results showed that addition of exogenous CoQ_10_ at either 50 μM or 100 μM showed no significant effect on lung cancer cell proliferation (Figures 7a,7b) and migration (Figures 7c, 7d). Therefore, the results highly suggested that the tumor-suppressing effects caused by PDSS2 in lung cancer cells might be independent on CoQ_10_ biosynthesis.

### PDSS2 suppresses lung cancer cell malignant features independent of its catalytic activity

**Figure 8:**
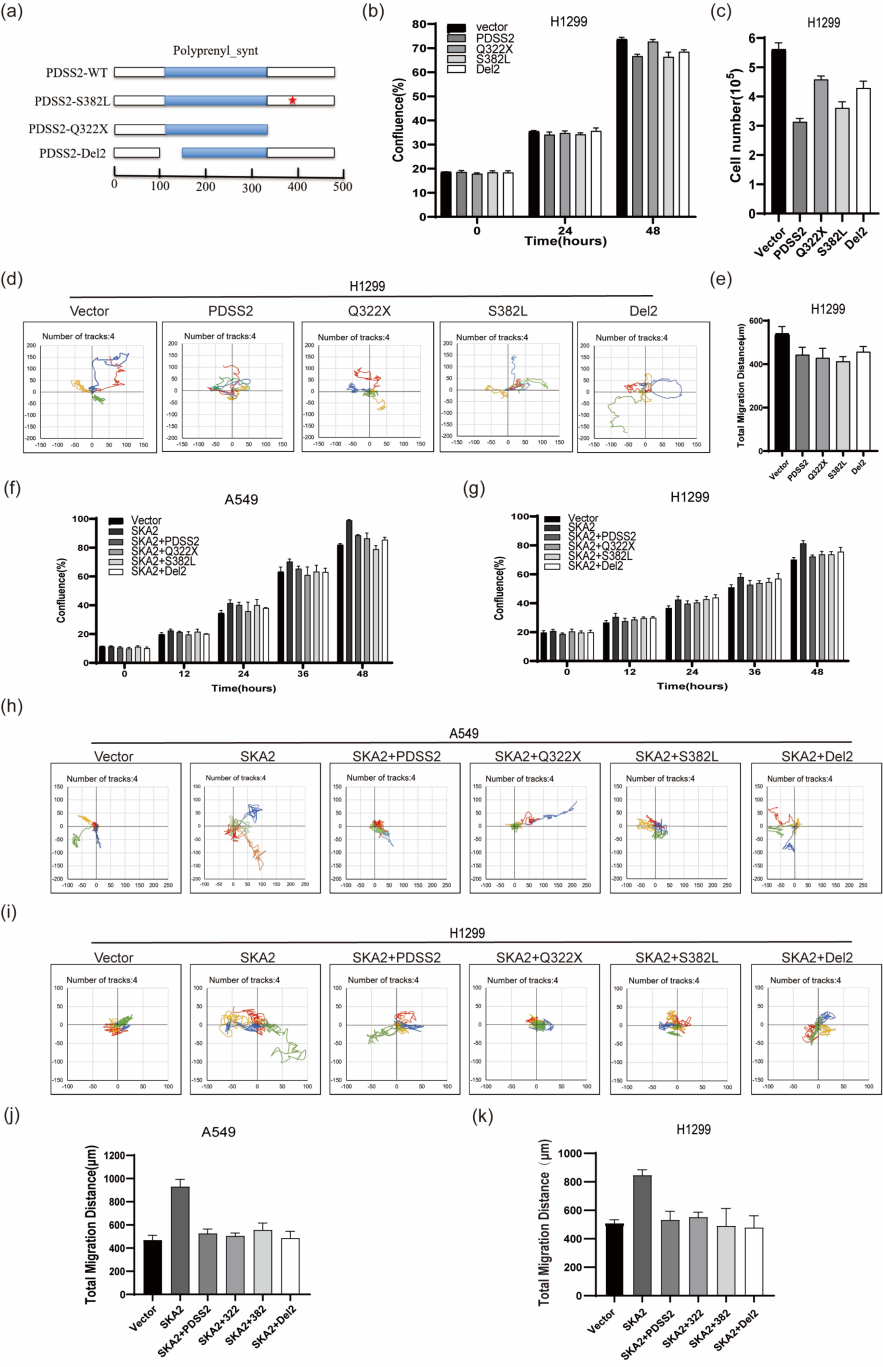
PDSS2 suppresses lung cancer cell malignant phenotypes independent of its catalytic activity. (**a**) Schematic diagram of the PDSS2 mutants structure, the scale means amino acids number. (**b,c**) PDSS2 mutants inhibited lung cancer cells proliferation. Cells were transfected with indicated plasmids and cell growth was continuously monitored by JULI Stage Real-time Cell History Recorder, and cell numbers were counted using CellDrop FL Fluorescence Cell Counter at the end of time-points. (**d,e**) PDSS2 mutants repressed lung cancer cells motility. Cell motility was also captured every 15 minutes for 12 hours by the aforementioned instrument. Motile trajectory, mean total migration distances and mean migration speeds are presented. (**f,g**) PDSS2 and its mutants abrogates SKA2-promoted A549 cells and H1299 cells growth. (**h,i**) Cell motility analysis. Cell trajectories showed that PDSS2 mutants abrogated SKA2-mediated promoting lung cancer cells motility. (**j,k**) The histogram showed the average distance of movement of A549 cells and H1299 cells, respectively.

As supplement of CoQ_10_ was unable to affect the malignant features in lung cancer cells, we speculate that PDSS2 might inhibit the lung cancer cell growth and motility independent of its catalytic activity of CoQ_10_ biosynthesis. Previous studies reported that a C-T transition at nucleotide 964 of PDSS2 coding region causes the mutation of amino acid 322 from glutamine to a stop codon, and C-T transition at nucleotide 1145 of PDSS2 coding region causes the mutation of amino acid 382 from serine to leucine (called respectively PDSS2-Q322X, PDSS2-S382L), which lead to significant decrease in CoQ_10_ biosynthesis 32. PDSS2-Del2, in which alternative splicing deleted exon2, is incapable of changing CoQ_10_ levels in cells with ectopic expression 33. Therefore, these three PDSS2 mutants were constructed (Figure 8a). The three PDSS2 mutants were transiently transfected into lung cancer cells, and the results clearly showed that all the three PDSS2 mutants showed obvious inhibitory effects on the growth and motility capabilities of lung cancer cells comparable to the wildtype PDSS2 construct (Figures 8b-8e). Of note, the results further showed that all the PDSS2 mutants could also abrogate the accelerated cell growth and motility mediated by SKA2 overexpression in both cell lines (Figures 8f-8k). Taken together, these findings strongly suggest that PDSS2 suppressed lung cancer cell malignant features independent of its catalytic activity of CoQ_10_ biosynthesis.

## Discussion

Lung cancer is one of the most prevalent cancers with high morbidity and mortality rates around the world. Researches are urgently eager to explore multiple effective therapeutic strategies for lung cancer. Our group previously shown that two novel potential oncogenic genes, PRR11 (proline-rich protein 11) and SKA2, play critical role in cell cycle progression, and accelerate lung cancer cell proliferation, motility and invasion 12, 25, 34, 35. And the two genes shared a bidirectional promoter negatively modulated by p53 through a transcription factor NF-Y in lung cancer cells 12, 17. In this study, we further investigated the molecular mechanisms underlying the implication of SKA2 in lung cancer progression. We found that PDSS2 is a novel downstream target of SKA2. SKA2 remarkably repressed PDSS2 gene expression at both mRNA and protein levels. SKA2 repressed PDSS2 promoter activity through its Sp1-binding sites. In addition, SKA2-mediated lung cancer cell aberrant proliferation and migration can be abrogated by exogenous overexpression PDSS2. Taken together, these findings elucidated that SKA2 promotes lung cancer cell progression at least partially via repressing PDSS2 expression.

PDSS2 is the critical enzyme implicated in CoQ_10_ biosynthetic pathway, and its function is to add isopentenyl diphosphates to geranylgeranyl diphosphate, thus producing the isoprenoid side chain 27-29, 32. PDSS2 mutations result in CoQ_10_ deficiency and then cause metabolic and nephritic diseases 32, 36-38. Previous studies have shown the relative importance of ROS (Reactive Oxygen Species) production and apoptosis in the pathogenesis of CoQ_10_ deficiency 39, 40. Here, our results showed that the level of CoQ10B expression was inhibited by SKA2. In contrary to our expectation, exogenous CoQ_10_ treatment showed no obvious effects on lung cancer cell growth and motility.* PDSS2* mutant fibroblasts do not show increased ROS production 40. In the present study, our results showed that all the three PDSS2 mutants showed comparable inhibitory effects on the growth and motility capabilities in lung cancer cells. This suggested that the tumor-suppressing effects caused by PDSS2 in lung cancer cells might be independent on CoQ_10_ biosynthesis. Therefore, PDSS2 might exert its function through its non-enzymatic function, e.g. interacting with other proteins. This speculation warrants further deep investigations.

Recent studies have shown that downregulation of PDSS2 gene is found in multiple cancers, such as gastric cancer, hepatocellular carcinoma, melanoma, and lung cancer. Lowered expression of PDSS2 enhances cancer cell proliferation and migration, and is closely correlated with reduced survival of cancer patients, implicating that PDSS2 will be a novel potential tumor suppressor gene 31, 33, 41-43. In this paper, suppressed lung cancer cell malignant phenotype by exogenous overexpression of PDSS2 was observed. And the PDSS2 expression was decreased in lung cancer tissues than in normal samples. Therefore, PDSS2 might serve as a potential novel target gene for diagnose and/or treatment lung cancer patients.

We and others previously reported that PDSS2 expression is inhibited by transcription factor Sp1 and DNA methylation modification in cancer cells 24, 41, 44. The expression of Sp1 in most cancers was higher than in para-carcinoma tissue, and high expression of Sp1 in cancers promotes cancer cell proliferation, migration, and invasion by regulating a variety of genes participating in tumorigenesis 45. Our previous study also found that Sp1 could directly mediate the transcription of PDSS2 in lung cancer cells 24. In this study, our luciferase reporter assay suggested that Sp1-binding sites in PDSS2 core promoter are required for SKA2-mediated repression of PDSS2 transcription. Co-immunoprecipitation assay demonstrated that SKA2 associated with Sp1. Therefore, it is possible that SKA2 could indirectly and/or directly interact with Sp1 to repress the transcription of PDSS2.

The glucocorticoid (GC) receptor (GR) is a member of the nuclear receptor superfamily of ligand-regulated transcription factors 46, 47. GR is capable of both upregulating and downregulating target gene expression 48 and inhibits cell cycle progression and cell proliferation in human lung cancer cell lines 49. Previous studies have demonstrated that SKA2 co-localized with GR in cell nuclei, and regulated cancer cell proliferation 8. We also found GR binding sequences on the PDSS2 promoter region (unpublished data). Therefore, further detailed studies are needed to investigate that whether SKA2 can also represses PDSS2 transcription through association with GR.

## Conclusions

**Figure 9:**
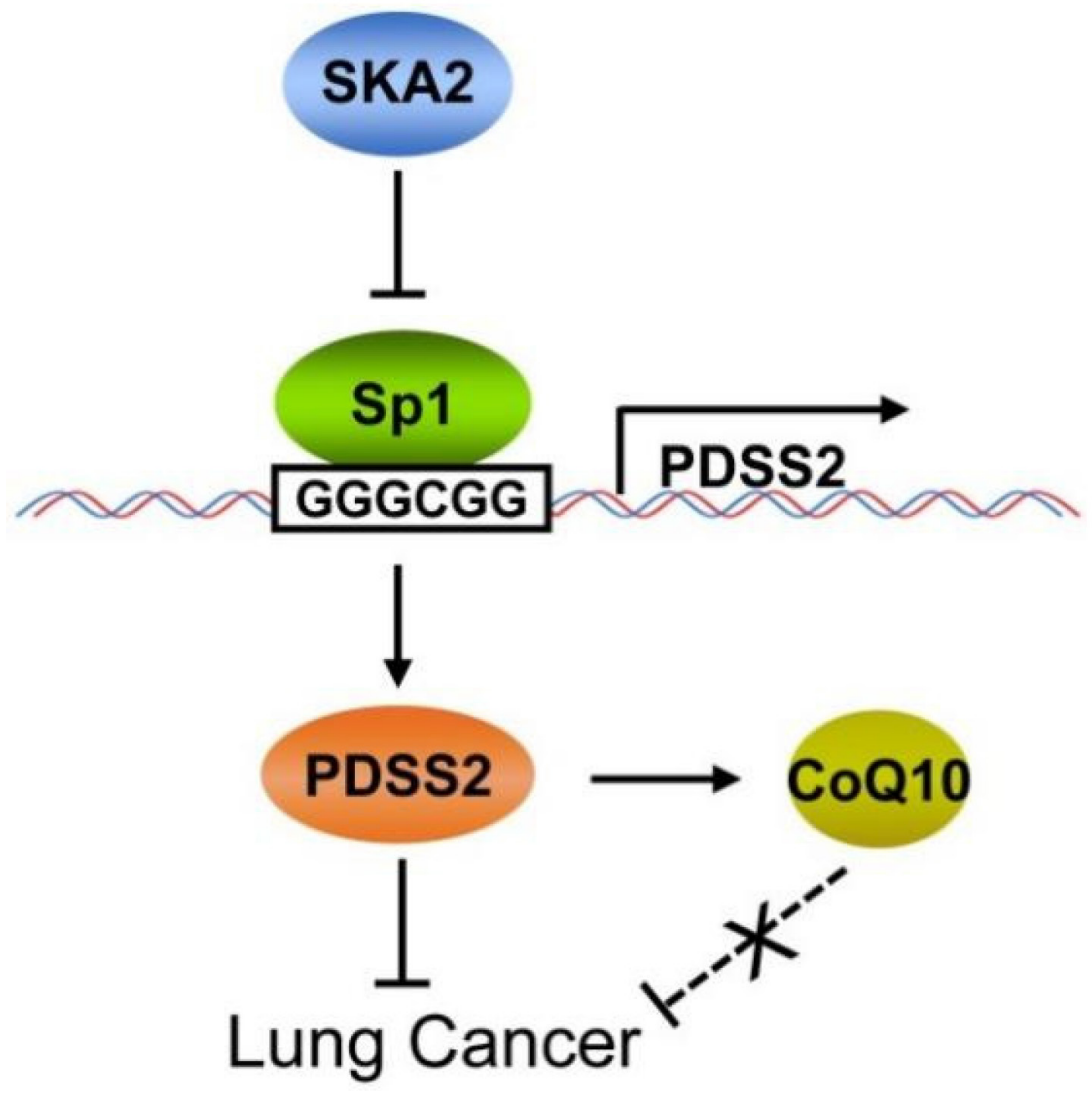
Working model for SKA2-mediated transcriptional downregulation of the key enzyme of CoQ10 biosynthesis PDSS2.

In conclusion, this study clearly demonstrated that PDSS2, the first key enzyme for CoQ_10_ biosynthesis, is a novel downstream transcriptional target of SKA2 in lung cancer cells, and SKA2 represses PDSS2 transcription through the Sp1-binding sites in PDSS2 core promoter. PDSS2 remarkably suppresses lung cancer cell growth and motility whereas CoQ_10_ has no obvious effects on lung cancer cell growth and motility, suggesting a non-enzymatic tumor-suppressing activity of PDSS2 in lung cancer cells (Figure 9). This novel transcriptional regulatory axis of SKA2 and PDSS2 functionally contributes to human lung cancer progression and is of potential prognostic and therapeutic significances for lung cancer patients.

## Figures and Tables

**Table 1:** The primer sequences used for overexpression vector construction and RNAi

Name	Sequence
pcDNA3.0-SKA2	Sense: 5'-GCGGCCGCGTCGACTCTCGAGATGGAGGCGGAGGTCG-3';
	Antisense: 5'-TATAGAATAGGGCCCTCTAGATCATAAATCTGGCATGTG -3'.
pcDNA3.0-Flag-PDSS2	Sense: 5'-GCGGCCGCGTCGACTCTCGAGACCATGAACTTTCGGC-3';
	Antisense: 5'-TATAGAATAGGGCCCTCTAGATCATGAAAATCTGGTCAC -3'.
pcDNA3.0-PDSS2Q322X	Sense: 5'-GAAGAGATTTGTGGATTAAAC-3'
	Antisense: 5'-CAAGAAATTCCTAATGTAAGAC-3'
pcDNA3.0-PDSS2S382L	Sense: 5'-GATCTGCTTTAGAAAACATTG-3'
	Antisense: 5'-TGGCCTCCAAGGGAGGAAAGC-3'
pcDNA3.0-PDSS2-Del2	Sense: 5'-TCAAAGAAGTTTGGCAGAGATC-3'
	Antisense: 5'-CTGGCTGTGGTAAGCAGAGG-3'
SKA2-siRNA-525	Sense: 5'-GGCUGGAAUAUGAAAUCAA TT-3'
	Antisense: 5'-UUGAUUUCAUAUUCCAGCC TT-3'
NC-siRNA	Sense: 5′-UUCUCCGAACGUGUCACGU TT-3′
	Antisense: 5′-ACGUGACACGUUCGGAGAA TT-3′
PDSS2-shRNA	Sense: 5'-GATCCGGAGACTTTCTTCTAGCAAATCAAGAGTTTGCTAGAAGAAAGTCTCTTTTTTGG-3';
	Antisense: 5'-AATTCCAAAAAAGAGACTTTCTTCTAGCAAACTCTTGATTTGCTAGAAGAAAGTCTCCG-'3.
NC-shRNA	Sense: 5′-TCATTCTCCGAACGTGTCACGTCTCGAGACGTGACACGTTCGGAGAATGTTTTTTC-3′;
	Antisense: 5′-TCGAGAAAAAACATTCTCCGAACGTGTCACGTCTCGAGACGTGACACGTTCGGAGAATGA-3′.

**Table 2:** The primer sequences for qRT-PCR

Names	Primer sequences
LOXL2	F451: 5'-ACTTTGTATGCCCGCTTTAA-3'
	R608: 5'-GCCGCAGTTTTCTCTTCTTT-3'
ABCA1	F4384: AACAGTTTGTGGCCCTTTTG
	R4540: AGTTCCAGGCTGGGGTACTT
IL6ST	F2716: TGTAGATGGCGGTGATGGTA
	R2961: CCCTCAGTACCTGGACCAAA
RGS17	F493: TTCTGGCTTGCTTGTGAAGA
	R661: GAGGATTGGGATCCAACAGA
CCL2	F162: CCCCAGTCACCTGCTGTTAT
	R332: TGGAATCCTGAACCCACTTC
CASP7	F550: AGTGACAGGTATGGGCGTTC
	R713: CGGCATTTGTATGGTCCTCT
LCOR	F1122: CTCTTGGGCAAAACCACATT
	R1349: GTGCGAGACTCCCAAGAAAG
PDSS2	F875: TCTAGCAAATGCCTGCAATG
	R1041: TCTGCTCCTTCCAAGTCGAT
GAPDH	F833: ACCTGACCTGCCGTCTAGAA
	R1060: TCCACCACCCTGTTGCTGTA
